# Pertussis vaccination in Child Care Workers: room for improvement in coverage, policy and practice

**DOI:** 10.1186/1471-2431-12-98

**Published:** 2012-07-13

**Authors:** Kirsty Hope, Michelle Butler, Peter D Massey, Patrick Cashman, David N Durrheim, Jody Stephenson, April Worley

**Affiliations:** 1 Hunter New England Population Health, University of Newcastle, Locked Bag 10, Wallsend, NSW, Australia, 2287; 2 Hunter New England Population Health, University of New England, Locked Bag 9783, NEMSC, Tamworth, NSW, Australia, 2340; 3 Hunter New England Population Health, PO Box 448, Forster, NSW, Australia, 2428

## Abstract

**Background:**

The “Staying Healthy in Child Care” Australian guidelines provide for illness and disease exclusions and encourage vaccination of staff in child care settings, however these requirements are not subject to accreditation and licensing, and their level of implementation is unknown. This study aimed to describe pertussis vaccination coverage in child care workers in a regional area of northern NSW during 2010; review current staff pertussis vaccination practices; and explore barriers to vaccination.

**Methods:**

A cross sectional survey of all child care centre directors in the Hunter New England (HNE) area of northern NSW was conducted in 2010 using a computer assisted telephone interviewing service.

**Results:**

Ninety-eight percent (319/325) of child care centres identified within the HNE area participated in the survey. Thirty-five percent (113/319) of centres indicated that they had policies concerning respiratory illness in staff members. Sixty-three percent (202/319) of centres indicated that they kept a record of staff vaccination, however, of the 170 centre’s who indicated they updated their records, 74% (125/170) only updated records if a staff member notified them. Of centres with records, 58% indicated that fewer than half of their staff were vaccinated.

**Conclusion:**

Many childcare workers have not had a recent pertussis immunisation. This potentially places young children at risk at an age when they are most vulnerable to severe disease. With increasing use of child care, national accreditation and licensing requirements need to monitor the implementation of policies on child care worker vaccination. Higher levels of vaccination would assist in reducing the risk of pertussis cases and subsequent outbreaks in child care centres.

## Background

The resurgence of pertussis (whooping cough) in Australia has attracted community concern, especially with recent deaths in two infants from the Australian state of New South Wales (NSW) [1,2]. Although pertussis incidence declined after the widespread use of whole cell pertussis vaccines in the mid-1940’s, this disease remains an important cause of morbidity in Australia, especially in young infants [3].

This bacterial infection of the respiratory tract, caused by *Bordetella pertussis,* usually begins with coryza (nasal conjestion), fatigue and sometimes a mild fever. A cough then develops, which is often paroxysmal, may be followed by a deep gasp (or whoop). Pertussis affects people of all ages with infants being at greatest risk of severe disease, complications, hospitalisation and death [4].

Pertussis notifications have recently increased in NSW, with averages rates for 2008/2009 (152.1 per 100,000 per year) being 2.7 times higher than the previous five year average (56.6 per 100,000 per year 2003/2007). The highest rates were reported in children less than five years of age (an average of 453 per 100,000 per year 2008/2009). Rates of hospitalization also increased during this period with a rate 3.1 times higher than the previous five year average (7.5 per 100,000 per year for 2008–2009, 2.4 per 100,000 per year for 2003/2007). Children aged 0–1 years of age experienced the highest hospitalisation rates (151.6 per 100,000 per year 2008/2009) [3].

Outbreaks in child-care setting are common and can be difficult to control. Outbreaks in these settings can result in many sick children, widespread antibiotic use in at-risk contacts with potential antibiotic adverse events [5].

In Australia there are a number of child care options, the most common are long day care centres and preschools. Long day care centres provide all-day care for children under school age. Preschool centres provide care for children from three years to five years of age and provide an introduction to class room learning [6].

In 2004, Australian children attending long day care did so for an average of 19 hours per week. By 2009 this had increased to an average of 26 hours per week [7]. The Australian Bureau of Statistics survey of families found that the proportion of Australian children attending formal care in the previous week increased from 17% in 1999 to 22% in 2008. In 2008, 9% of the population of children aged less than 12 months were in formal child care. The proportion attending formal care peaked at 50% at three years of age, after which it declined to 20% by age five [7].

In Australia there are recommendations for the immunisation of childcare workers [8] but there is no supportive funding for vaccines or vaccine administration, except in NSW were at the time of the study free vaccines were available for individuals who care for children under the age of one year and is now free only for mothers of newborns. Previous research has shown that a high proportion of staff take leave due to infectious diseases [9] and that staff immunization is vital for maintaining a safe environment for children [10,11].

In Australia there are guidelines, the “Staying Health in Child Care” manual, which outline procedures that should be adhered to in child care centres to ensure children stay healthy. Amongst other measures, immunisation of children and staff is also recommended. The manual indicates that “Child care staff may be exposed to diseases that are preventable by immunisation, including hepatitis A, measles, mumps, rubella, varicella and pertussis. Immunisation of staff is one effective way to manage the risk in childcare settings, as these diseases are usually infectious before the onset of symptoms” [12]. Although these guidelines provide for exclusions and encourage vaccination, they are not supported by accreditation [13] and licensing requirements [14]. The level of compliance to these guidelines in Australia is not known.

The aim of this project was to describe pertussis vaccination coverage in child care workers in child care centres located in a regional area of northern NSW during 2010. The project also explored pertussis vaccination policies and barriers to vaccination for staff.

## Methods

### Study design

A cross sectional survey of all child care centre directors in the Hunter New England (HNE) region of northern NSW was conducted in September and October 2010. This regional area covers both rural and metropolitan areas, with a population of approximately 875,000 including 57,600 children under five years of age and 325 child care centres [15].

Child care centre names (including long day care and preschools), and contact details were obtained from the Department of Community Services child care centre database [16]. Ethics approval for the study was obtained from the Hunter New England Human Research Ethics Committee.

An introductory letter was sent to all 325 child care centre directors providing a brief background to the project. Subsequently a trained interviewer contacted each director by telephone, up to six call backs were made if telephone contact was unsuccessful. Directors were asked to complete a short telephone survey (appropriately 10 minutes in duration) via the computer assisted telephone interview (CATI) service. The CATI process uses an electronic survey (as defined below), where the trained interviewer can enter the answers from the respondents immediately into a SAS 9.2 (SAS Institute, Carry, NC, USA) database. At the conclusion of the interview, information was sent to directors concerning staff vaccination recommendations and material to help develop a system for staff vaccination. In addition the NSW Health fact sheet on pertussis vaccination was distributed after the interview for use as a staff information fact sheet.

### Survey content

The survey instrument consisted of questions designed to obtain information in three areas: centre characteristics, staff vaccination coverage and barriers to staff vaccination.

Centre characteristic questions included staff numbers, registered child places, age split of rooms and business model (single business compared to franchises). Vaccination coverage included number of staff vaccinated, staff vaccination records and staff vaccination policies. Barriers to vaccination explored included: reasons why staff had not been vaccinated and strategies available to increase vaccination. Barrier to vaccination questions included optional responses and to specify if the response was not given. The survey instrument was piloted with two child care centre directors.

### Statistical methods

Analysis was conducted using SAS 9.2 (SAS Institute, Carry, NC, USA), including frequencies and Chi square tests for differences in proportions.

## Results

### Response rate and sample characteristics

Of the 325 child care centres registered within the Hunter New England area, 319 (98.2%) completed the survey, 5 declined to participate and 1 could not be contacted. Of the 319 centres participating in the survey, 63% (n = 201) were long day care centres and 37% (n = 118) were preschools. Sixty eight percent (n = 217) of the centres were located within urban areas and 83% (n = 266) had a stand-alone business (Table 1). Seventy-six percent (153/201) of long day care centres looked after children under one year of age and all centres looked after children two years or older. All preschools looked after children three years and over. The median number of staff and size of the centre is outlined in Table 1.

**Table 1:** Characteristics of child care centres completing the pertussis vaccination survey, Hunter New England region, 2010

**Demographic**	**N**	**%**
**Facility Type**		
Long Day Care (LDC)	201	63 %
Preschool	118	37 %
**Area**		
Urban	217	68 %
Rural	102	32 %
**Ownership**		
Single centre	266	83 %
Multiple centres	53	17 %
**Size of facilities**		
	**Median**	**min-max**
No. Staff	10	2,54
No. Students	39	8,96
	**N**	**%**
1 room	115	36 %
2 rooms	97	30 %
3 or more rooms	107	34 %

### Respiratory Illness policies

Thirty five percent (113/319) of centres indicated that they had policies concerning respiratory illness in staff members. Long day care centres were more likely to have a policy than preschools (41%, 83/201 and 25%, 29/118, respectively, p = 0.002). When asked, directors indicated that 58% (66/113) of centres with a policy had general illness polices; 15% (17/113) indicated that staff required clearance from a GP to return to work after a respiratory illness, 14 % (16/113) indicated staff are told not to come to work if ill , 6% (7/113) indicated that they followed the “Staying Healthy in Child Care” guidelines in relation to staff illness and 6% (7/113) were not sure.

### Vaccination coverage

Sixty-three percent (202/319) of centres indicated that they kept some record of staff vaccination history. Long day care centres were more likely to keep records than preschools (84%, 168/201 and 28%, 33/118, respectively p < 0.0001). Eighty-four percent (170/202) of centres keeping records indicated that they updated these records. However 74% (125/170) indicated it was up to the staff member to notify them of any vaccinations, while 36% (61/170) indicated that they actively updated records annually. When asked specifically about pertussis vaccination, 59% (189/319) of centre directors indicated that they knew whether their staff were vaccinated against pertussis or not; with similar proportions for both types of centre.

**Figure 1:**
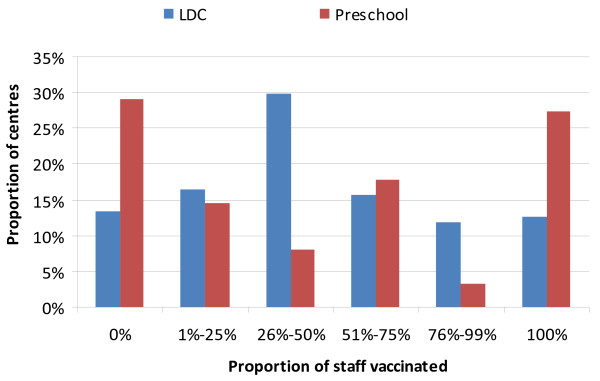
Proportion of Staff Vaccinated (for centres where numbers were available) by LDC and preschool, HNE, 2010.

Of the centre directors who indicated that they kept records of staff vaccination, 94% (189/202) indicated that they knew if staff were vaccinated against pertussis. Fifty-seven (117/202) percent of these centre directors indicated that less than half of their staff were vaccinated. Figure 1 indicates the coverage distribution by LDC and Preschool. Within the 319 centres there are 3574 carers of which 1050 (29.4%) were known to be immunised Thirty percent of LDC staff and 23% of preschool staff were reported to be vaccinated. All centres were keen to have information to assist them to keep adequate and practical vaccination records.

### Barriers to vaccination

Of the 319 centres interviewed 288 (90%) provided an answer for why their staff were not vaccinated. Sixty-seven percent, (194/288) of directors indicated that they had not asked or did not know why their staff were not vaccinated, while 16% (45/288) admitted not knowing that their staff should be vaccinated against pertussis. Directors indicated that to increase staff vaccination they needed to provide staff with information about vaccination (73%; 232/319), and that free vaccine and time to get vaccinated (69%; 219/319) would need to be organised. Having someone visit the centre to vaccinate staff (47%; 150/319) was considered potentially useful.

## Discussion

We found that over half (63%) of childcare centres in the Hunter New England area kept records of staff vaccination, and a large proportion of staff were not vaccinated against pertussis. Preschools were shown to be statistically less likely to keep records of staff vaccination. While those at greatest risk are infants less than one year of age, older children still experience morbidity and are a known source of infection for younger siblings [17,18].

Recent research conducted in Australia has shown that pertussis immunity following vaccination decreases by three years of age [19]. With 50% of three year olds in child care [7] and siblings being a key source of infection for children under one year of age [17,18], it is vital that they are protected from infection.

Excluding sick children and staff is an important way of limiting the spread of infection in a child care centre. Having a written policy that clearly states the centre’s exclusion criteria is also important. In this study less than half the centres had policies for respiratory illness in staff. Only 6% of centres mentioned the “Staying Healthy in Child Care” manual. A previous study is Australia in 2006 also identified low awareness of guidelines for staff immunisation [20]. In the four years since this study, the implementation of these guidelines has not changed and remains low.

Child care centres are required to keep up-to-date records of children’s vaccination status and to exclude those that are not vaccinated; however this has not been extended to include child care centre workers. Of particular concern was that 19% of centres knew that none of their staff were vaccinated against pertussis.

Two thirds of the directors did not know why staff had not had a pertussis vaccination, suggesting this had not been discussed with staff. A barrier to staff immunisation in some centres is likely to be that 16% of directors reported not knowing that their staff should be vaccinated against pertussis. Empowering directors with useful information about staff vaccination may result in more complete follow-up of vaccination status in staff, as is done for the children who attend centres. This could be done through requirement in accreditation or licensing processes.

A strategy to minimise transmission in these settings would be to enforce the guidelines in the “Staying Healthy in Child Care” manual for staff vaccination during accreditation or licensing processes. Guidelines include:

· develop a staff immunisation policy; which outlines the immunization requirements for childcare staff at the centre which are inline with NHMRC requirements;

· develop a staff immunisation record; this should document previous infection or immunisation for the relevant diseases;

· require all new and current staff to complete the staff immunisation record;

· regularly update staff immunisation records as staff become vaccinated;

· provide staff with information about diseases that are preventable by immunisation, for example through in-service training and written material such as fact sheets; and

· take all reasonable steps to encourage non-immune staff [12].

The “Staying Healthy in Child Care” manual is currently being reviewed by the NHMRC, with a new version scheduled for release in 2012.

This study highlighted the influence of the presence of a respiratory illness policy and vaccination coverage between long day care centres and preschools. Due to the age of the children (under 1 year of age) long day care centers are required to have a higher carer to child ratio then preschools. However both are required to have qualified staff depending on the number of children. Both types of day care are required to be licensed however not all preschools are accredited. Therefore the difference in policies and vaccination coverage may be due to different accreditation practices.

Even though this study had a very high participation rate there are a number of limitations. Child care centres in the regional area studied may not be representative of all child care centres in the state. The survey relied on information provided by the centre director not the individual child care workers and this could have been influenced by recall bias and time constraints in answering the questions. In addition, the questions relating to vaccination coverage were not open ended, directors were provided with options to choose from, therefore the categories of response could have been biased by the survey design. However the options were previously pilot tested with two child care centre directors in an attempt to reduce bias.

## Conclusion

Many childcare workers have not had a recent pertussis immunisation. This is potentially placing young children, who are most vulnerable to severe pertussis disease, at risk. Outbreaks of pertussis in child-care settings can be difficult to control, and can result in many sick children and widespread use of antibiotics. With increasing use of child care, national accreditation and licensing requirements should include the need for documented child care worker vaccination. Improving vaccination coverage of child care workers and good policy implementation monitoring will reduce the risk of pertussis outbreaks in child care centres.

## Competing interests

The authors declare that they have no competing interests.

## Authors’ contributions

KH lead the design, implementation, statistical analysis and manuscript development. MB participated in the design of the study, the statistical analysis and drafting the manuscript. PM conceived the study, participated in the design and drafting the manuscript. PC provided expert advice, participated in the design and drafting the manuscript. DD provided expert advice, participated in the design and drafting the manuscript. JS participated in the design and implementation of the project. AW participated in the design and implementation of the project. All authors read and approved the final manuscript.

## Pre-publication history

The pre-publication history for this paper can be accessed here:

http://www.biomedcentral.com/1471-2431/12/98/prepub
